# A Washing-Free and Easy-to-Operate Fluorescent Biosensor for Highly Efficient Detection of Breast Cancer-Derived Exosomes

**DOI:** 10.3389/fbioe.2022.945858

**Published:** 2022-06-28

**Authors:** Wenqin Chen, Yan Zhang, Kaili Di, Chang Liu, Yanyan Xia, Shijia Ding, Han Shen, Zhiyang Li

**Affiliations:** ^1^ Department of Clinical Laboratory, Nanjing Drum Tower Hospital, The Affiliated Hospital of Nanjing University Medical School, Nanjing, China; ^2^ Key Laboratory of Clinical Laboratory Diagnostics (Ministry of Education), College of Laboratory Medicine, Chongqing Medical University, Chongqing, China

**Keywords:** exosomes, fluorescence biosensing, aptamer, G4-hemin, tumor diagnosis

## Abstract

Traditional detection methods for protein tumor markers in the early screening of breast cancer are restricted by complicated operation procedures and unstable reproducibility. As one of alternative emerging tumor markers, exosomes play an important role in diagnosing and treating cancers at the early stage due to traceability of their origins and great involvement in occurrence and development of cancers. Herein, a washing-free and efficient fluorescent biosensor has been proposed to realize simple and straightforward analysis of breast cancer cell-derived exosomes based on high affinity aptamers and G quadruplex-hemin (G4-hemin). The whole reaction process can be completed by several simple steps, which realizes washing-free and labor-saving. With simplified operation procedures and high repeatability, the linear detection range for this developed fluorescent biosensing strategy to breast cancer cell-derived exosomes is from 2.5 × 10^5^ to 1.00 × 10^7^ particles/ml, and the limit of detection is down to 0.54 × 10^5^ particles/ml.

## Introduction

Breast cancer has been the number one killer of women’s health in recent years (Jordan, 2020; Houghton and Hankinson, 2021). Diagnosis of tumors usually relies on tissue biopsy in clinical, which requires very complex operation and may cause great harm to patients. Restricted by the complex operating procedures and instability of output results from current clinical detection methods, the reference value of marker proteins detection results, such as human epidermal growth factor receptor 2 (HER2) in the early screening of breast cancer has been reduced (Lamtha et al., 2021; Tang et al., 2021). Therefore, conceiving new detection strategies to make up for the deficiencies and achieve more effective early screening and diagnosis of breast cancer is an urgent problem. Liquid biopsy, as a promising approach, can detect tumor markers from blood, saliva, urine and other body fluids, avoiding the injury caused by surgical operation and puncture to patients. Current methods of liquid biopsy mainly include circulating tumor cell (CTC), circulating tumor DNA (ctDNA), exosomes, and extracellular free nucleic acids (Kilgour et al., 2020; Ahmadi and Rezaie, 2021). Exosomes (30–150 nm) are extracellular vesicles secreted into body fluids by various living cells and involved in signal transmission and regulation. In recent years, exosomes have gradually become one of the most popular biomarkers for liquid biopsy due to the fact that they contain a variety of genetic materials from parental cells and their high participation in the occurrence and development of various diseases (Li et al., 2019b; Shen et al., 2020). Cells are stimulated to secrete more exosomes, far more than in normal physiological states. Moreover, overexpressed tumor-specific proteins are also present on the surface of tumor cell derived exosome membranes (Liang et al., 2020; Rezaie et al., 2022; Vahabi et al., 2022). Tumor cell-derived exosome specific protein or RNA markers can be used to indicate tumor development and invasion degree. Compared to other liquid biopsy targets such as CTC and ctDNA, exosomes are more abundant and stable due to the vesicles protection, which benefit to overcome the problem of easy degradation (Ignatiadis et al., 2021). Thanks to all above characteristics, exosomes undoubtedly become one of the biomarkers worthy of in-depth study and have far-reaching significance for early screening, preventive diagnosis and treatment of tumors.

Researchers have conceived a number of strategies to detect tumor cell-derived exosomes based on various detection platforms, such as electrochemistry (Huang et al., 2019), electrochemiluminescence (Li et al., 2021b), immunoassay (Wang et al., 2021) and thermophoresis (Timson, 2019), all of which obtained satisfactory detection results. However, these methods often require complicated and time-consuming washing steps, which make it difficult to guarantee the integrity of exosomal membranes and the repeatability of the experiment. In addition, most of these methods need to be tested by professionals on expensive ancillary equipment, which further increases capital and labor costs. Therefore, it is necessary to propose a new detection method to make up for the deficiencies of existing methods and achieve simple, cheap and sensitive detection of exosomes. Fluorescence detection is a natural luminescence reaction with extremely high sensitivity and wide linear range (Zhang et al., 2019; Huang et al., 2020). As a benefit from this, the interference of non-fluorescent components can be considerably avoided, which is the optimum choice for most trace target analysis (Casto-Boggess et al., 2022). Traditional fluorescence detection methods mostly use ready-made peroxidases such as HRP (Lu and Xu, 2019; Heo et al., 2021) and ALP (Han et al., 2020; Wu et al., 2022) to catalyze the substrate such as tyramine (Zhang et al., 2020a; Kang et al., 2020) and thioflavin T (Wu et al., 2018; Pramanik et al., 2021) to output fluorescent signals. The disadvantages of this method lie in that the enzyme protein is relatively unstable and has harsh requirements on reaction conditions, which make researchers turn their attention to the G4-hemin biomimetic enzyme (Li et al., 2021a; Huang et al., 2021). The advantages of G4-hemin are excellent catalytic performance, high structural flexibility and stability (Cao et al., 2020; Chen et al., 2020). Moreover, Just like G4-hemin, aptamer is one of the functional nucleic acids that can bind with corresponding ligand with high affinity and strong specificity, and is also a frequent visitor in the field of biosensing research (Connelly et al., 2021; Daems et al., 2021; Nakatsuka et al., 2021).

Herein, we proposed a simple and easy-to-operate biosensing strategy for efficient detection of breast cancer cell-derived exosomes based on fluorescence platform. First, in this strategy, we combined the high specificity of aptamer with excellent catalytic performance of G4-hemin through sophisticated structural design of the bicyclic capture probe. In addition, the whole reaction process requires only streamlined sample addition and no complicated washing steps are needed, which greatly saves time and labor costs. The washing-free and simple operation steps realize the highly sensitive detection of breast cancer cell-derived exosomes, which not only solves the problem of low repeatability in traditional detection methods, but also reduces the requirements for equipment and has the potential for clinical promotion.

## Experimental

### Reagents and Materials

Fetal Bovine Serum (FBS) and Dulbecco’s Modified Eagle Medium (DMEM) were offered by Gibco (Gaithersburg, MD, United States, https://www.thermofisher.com). Phosphate buffer (PBS) was supplied by Thermo Fisher Scientific (Wilmington, United States, https://www.thermofisher.com). Monoclonal antibodies such as anti-CD63, anti-CD9 and anti-HER2 were obtained from Abcam (MA, United States, https://www.abcam.cn), and polyclonal antibody of HRP rabbit IgG were purchased from Beyotime (Jiangsu, China, https://www.beyotime.com). All of these antibodies were dissolved in 0.1% bovine serum albumin (BSA) solution. Hemin and tyramine were supplied by Sigma-Aldrich (St. Louis, United States, https://www.sigmaaldrich.com). We prepared dimethyl sulfoxide (DMSO) as stock solution to dissolve pure hemin, and then utilized HEPES (NaCl 200 mM, KCl 100 mM, DMSO 1%, Triton 0.05%) buffer solution to dilute the above hemin solution to different concentrations. Analytical reagent grade chemicals were utilized all through the experiment. As shown in Supplementary Table S1, all of the oligonucleotides were purified by high performance liquid chromatography. Next, the 30% hydrogen peroxide (H_2_O_2_) was supplied by Sangon Biotech. Co., Ltd. (Shanghai, China, https://www.sangon.com). We utilized the buffer of Tris-EDTA (TE) to dissolve and dilute all of the needed oligonucleotides for its suitable PH value and ion components. Lastly, we used the aqueous solutions supplied by the Millipore Milli-Q gradient ultrapure water system (Millipore Co., MA, United States, https://www.merckmillipore.com) all over the experiment.

### Instruments

Fluorescence detections were carried out on a Cary Eclipse Fluorescence Spectrophotometer (Agilent Technologies, United States, https://www.agilent.com). Transmission Electron Microscope (TEM) image, Nanoparticle Tracking Analysis (NTA), the SDS-PAGE and gel imaging analysis for the characterization of tumor cell-derived exosomes was supported by H-7500 transmission electron microscope (Hitachi High-Technologies Co., Japan, https://www.hitachihightech.com), ZetaView (Particle Metrix, Germany, https://www.particle-metrix.com), electrophoresis analyzer and ChemDoc XRS (Bio-Rad, United States, https://www.bio-equip.com), respectively.

### Extraction Procedures for Exosomes

We obtained several needed tumor cell lines as the control subjects, such as SK-BR3, HeLa, MCF-7, LNCaP and HepG2 from the American Type Culture Collection (ATCC) (Rockville, United States, https://www.atcc.org). After addition of 1% streptomycin and penicillin and 10% FBS, the cell culture medium named Dulbecco’s Modified Eagle Medium (DMEM) was used all over the experiment for tumor cell culture. All cells were cultured in a sterile environment throughout the whole process, and appropriate timing was selected for passage and cryopreservation according to the speed of cell growth and reproduction. We observed the growth state of the cells and when they grew well and covered about 80% of the culture dish, and then we reduced the nutrients from the cells and performed starvation culture. When the cells propagated in serum-free medium for 48 h, we proceeded to the next process of extracting exosomes. The cells were stressed in a nutrient-deficient growth environment and secreted far more exosomes than normal cells, so we got increased production of exosomes. Then we started the extraction process for exosomes (Zhang et al., 2020b). First, the cell culture medium for starvation culture was collected and centrifuged (10,000 ×g, 30 min) for preliminary purification to remove impurities such as cell debris with a large mass. The collected supernatant was then subjected to ultracentrifugation (100,000 ×g, 70 min) twice to continue purification of exosomes. It was critical to resuspend the pellet between the intervals of twice ultracentrifugation. After the first ultracentrifugation, the supernatant was discarded. Then, after adding 1 × PBS solution, the precipitate was resuspended before the second ultracentrifugation. Then, after the second ultracentrifugation, the supernatant was also discarded, and 100 μl 1 × PBS was added to resuspend the obtained exosome pellet. The step for extracting exosomes from clinical serum samples required an additional procedure on the basis of the above process. In general, the final exosome solution needed to be passed through a 0.22 μm filtration membrane to minimize the interference of other free-floating proteins and extracellular vesicles. The whole extraction process was carried out at 4°C to prevent exosome degradation. Finally, the extracted exosomes were characterized by TEM, NTA and western blot, and then stored at −80°C. The clinical samples were collected from the Affiliated Drum Tower Hospital of Nanjing University Medical School.

### Fluorescent Biosensing of Exosome

For exosome recognition, 2 μl target exosome with various concentrations was added to a 97 μl reaction mixture containing 165 nM of G4 hairpin capture probe, and incubated at 37°C for 60 min. Then, hemin (98 μl, 154 nM), tyramine (2 μl, 160 mM), and freshly prepared H_2_O_2_ (1 μl, 200 mM) was added to the reaction mixture and reacted at room temperature for 10 min for G4-hemin biosensing. Finally, we scanned the above reaction products with a voltage of 600 V, and obtained the fluorescence emission spectrum between 330 and 500 nm under excitation wavelength of 320 nm.

## Results and Discussion

### Design of Fluorescence Biosensor

**SCHEME 1:**
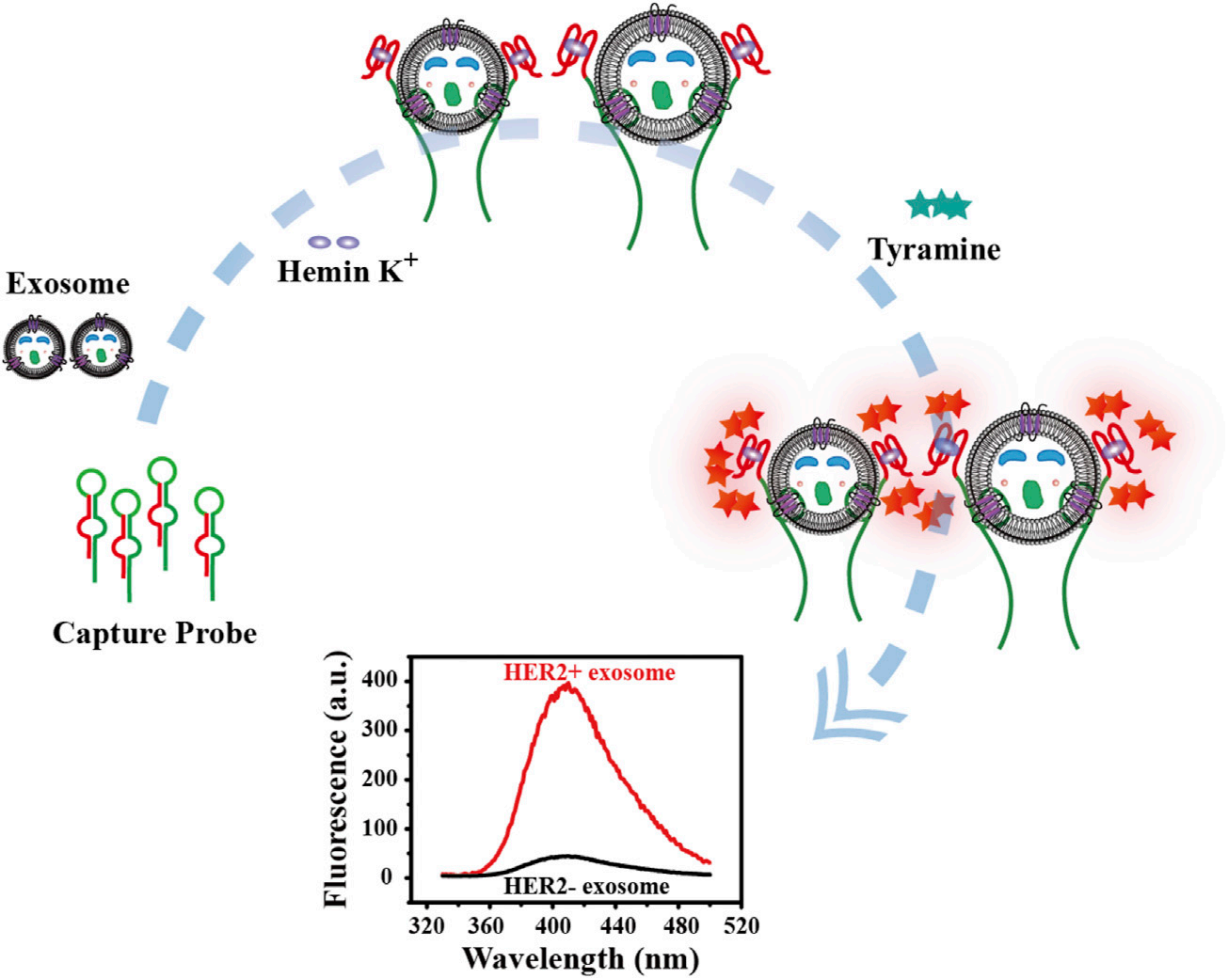
Schematic diagram of principle for detection of tumor cell-derived exosomes.

The principle for detecting breast cancer cell-derived exosomes is shown in Scheme 1. First, the bicyclic capture probe was obtained by heating and annealing. The exosomes used in the experimental verification stage were extracted from cultured tumor cells by traditional ultracentrifugation. Different concentrations of exosomes were added to the capture probe solution, and the double rings were opened by the high affinity between the aptamer and HER2 protein over-expressed on the membrane of the SK-BR3-derived exosomes (Liu et al., 2019; Vajhadin et al., 2022). The terminal G4 sequence was exposed while tightly capture exosomes. After full reaction, hemin was added to generate G4-hemin catalytic enzyme. The best catalytic state was achieved by adjusting the optimal concentration ratio. Then, the addition of tyramine incited the catalysis of G4-hemin to output a fluorescent signal (Li et al., 2019a; Wang et al., 2020). Quantitative detection of exosomes was achieved by the fluorescence intensity. The highlight of this strategy is integration of target recognition, capture and signal output, which is reasonably reflected in the ingeniously designed bicyclic capture probe. More importantly, the washing-free and streamlined sample addition steps ensure the facile and efficient detection of breast cancer cell-derived exosomes.

### Characterization of Exosomes

**FIGURE 1:**
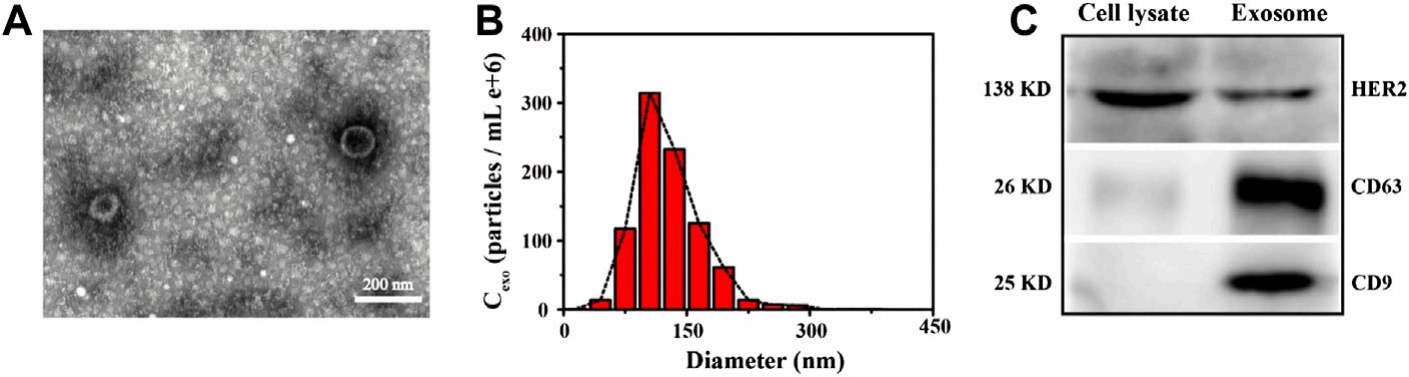
Characterization of SK-BR3 cell exosomes by TEM **(A)**, NTA **(B)** and Western blot **(C)**, respectively. Scale bars = 200 nm. Cell lysate was set as a reference.

We chose Transmission Electron Microscope (TEM), Nanoparticle Tracking Analysis (NTA) and western blot to characterize the appearance, morphology and particle size distribution of the extracted breast cancer cell-derived exosomes, as shown in Figure 1. It can be seen from the TEM image on the left (Figure 1A) that the extracted exosomes were circular or elliptical vesicles with a double layer membrane. Meanwhile, we learned from the NTA characterization graph (Figure 1B) that the size of the extracted exosomes was mostly distributed between 75 and 200 nm, concentrated around 100 nm, which was consistent with information reported in the literatures (Le and Fan, 2021; Zhang et al., 2021). In addition, taking an authoritative literature as a guideline (Théry et al., 2018), we also performed western blot experiments to characterize common markers (CD63, CD9) and the specific marker HER2 on the membrane of SK-BR3 cell-derived exosomes. It can be seen from Figure 1C that these proteins are enriched in the target exosome membrane, which indicates the successful extraction process and supports the reliability of subsequent experiments.

### Feasibility of Developed Biosensor

**FIGURE 2:**
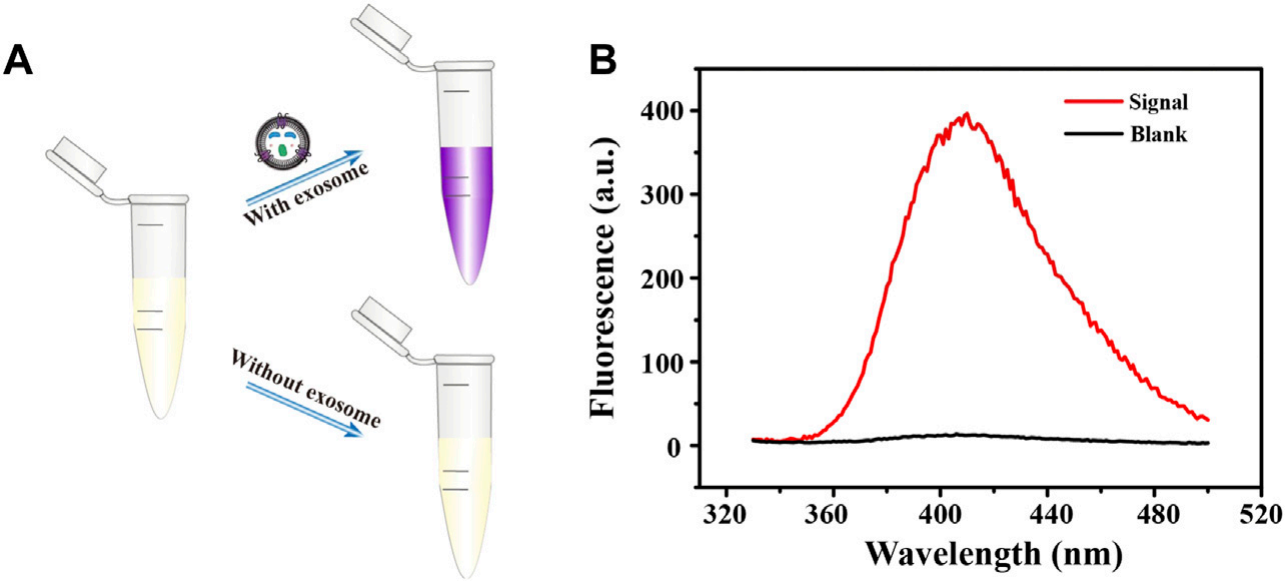
Brief schematic **(A)** and fluorescence emission spectra **(B)** of fluorescent biosensing protocol in the detection of exosomes.

We then further verified the feasibility of this biosensor. As shown in Figure 2, the color of the solution changed only when the exosomes were present, from colorless to purple (Figure 2A), and a strong fluorescent signal was produced (Figure 2B). By contrast, the color of the blank control did not change and the fluorescence signal was almost negligible, which indicates that only the target exosomes can open the bicyclic capture probe to release the G4 sequence. Thanks to the high specificity of aptamer and excellent catalytic performance of G4-hemin, the facile and sensitive detection of breast cancer cell-derived exosomes was achieved successfully. At the same time, the washing-free and simple operation procedure improved the reproducibility and repeatability of this detection method, broadening the future application range.

### Optimization of Experimental Parameters

**FIGURE 3:**
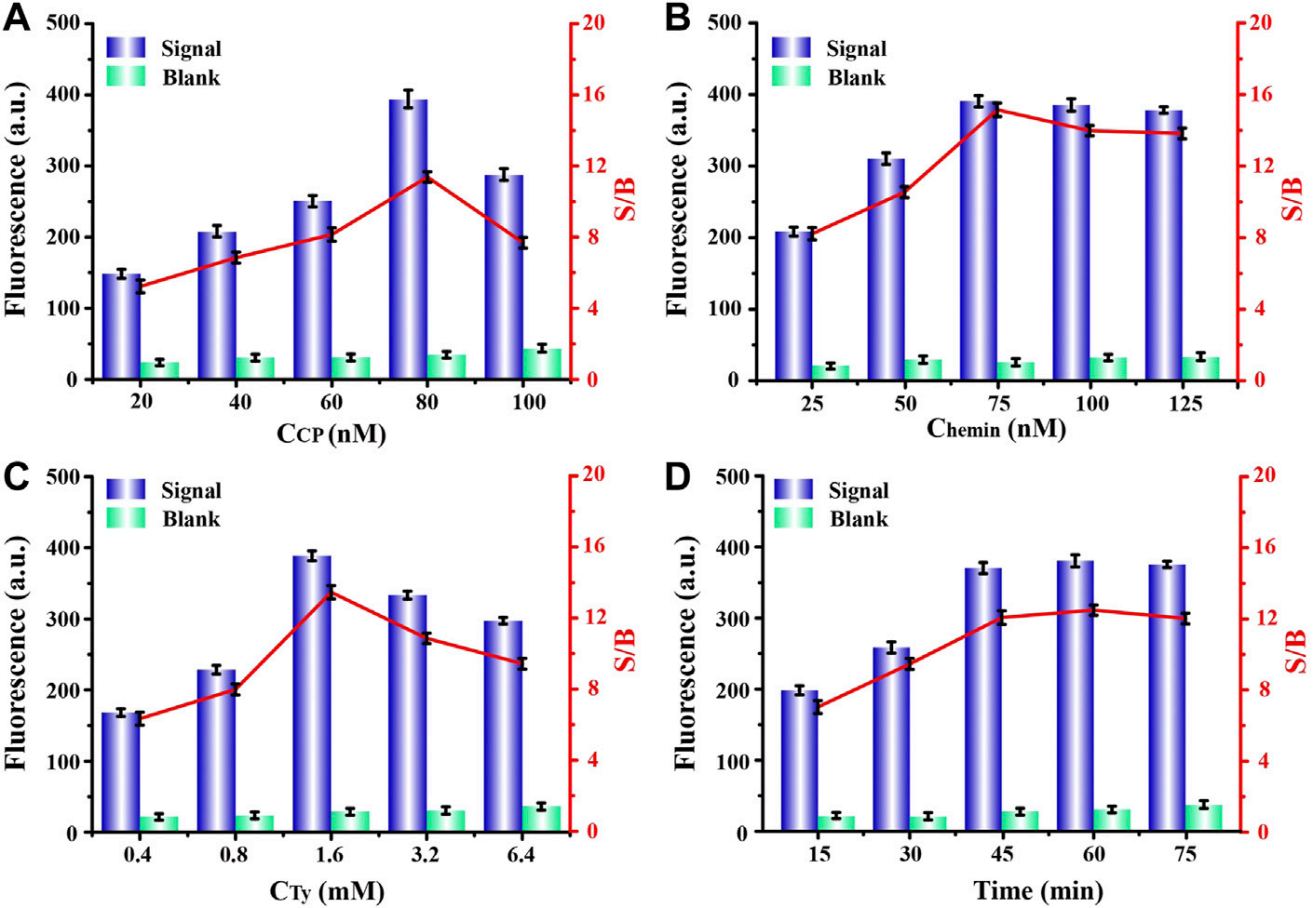
Optimizations of experimental concentrations of **(A)** capture probe, **(B)** hemin, **(C)** tyramine, and **(D)** required reaction time for capture probe and exosomes, respectively.

In order to achieve the best experimental results, we optimized a series of experimental conditions. First, the bicyclic capture probe was one of the most important components in this program, and its concentration had a significant impact on the signal output. Thereafter, we compared the fluorescence signals obtained from experiments using a range of probe concentrations. By observing Figure 3A, we can see that, when the probe had a concentration of 80 nM the obtained fluorescence signal was highest, and the signal-to-noise ratio also reached the maximum. When the concentration of the capture probe was further increased, the gradually enhanced steric hindrance limited the catalytic efficiency of G4-hemin to a certain extent, resulting in a decrease in the fluorescence intensity. Therefore, we chose 80 nM as the capture probe’s optimal concentration for follow-up experiments. Next, the influence of hemin concentration cannot be ignored. The existence of hemin directly determines the formation and catalytic performance of G4-hemin (Ahmadi et al., 2021), which is an important key point of signal output in this scheme. It can be seen from Figure 3B that, only the 75 nM concentration of hemin obtained the highest signal and largest signal-to-noise ratio, so it was selected as suitable concentration. Additionally, in order to provide a resource-rich environment for maximum catalytic effect of G4-hemin, it was necessary to optimize the concentration of reaction substrate tyramine. We learned from Figure 3C that, too low tyramine concentration cannot meet the substrate requirements of the catalytic reaction, while too high tyramine concentration will also have a certain inhibitory effect on the catalytic reaction of G4-hemin. Therefore, according to the experimental results, to achieve the best signal output, the concentration of tyramine should be controlled to 1.6 mM. Finally, we also optimized the reaction time after adding the exosomes. It can be seen from Figure 3D that, when the reaction time was 60 min, the fluorescence signal and signal-to-noise ratio were both the highest, but when the reaction time was set for 45 min, the signal and signal-to-noise ratio obtained were hardly the same as the best state. After weighing the pros and cons, choosing a reaction time of 45 min was more efficient and time-saving.

### Analytical Performance of Fluorescent Biosensor

**FIGURE 4:**
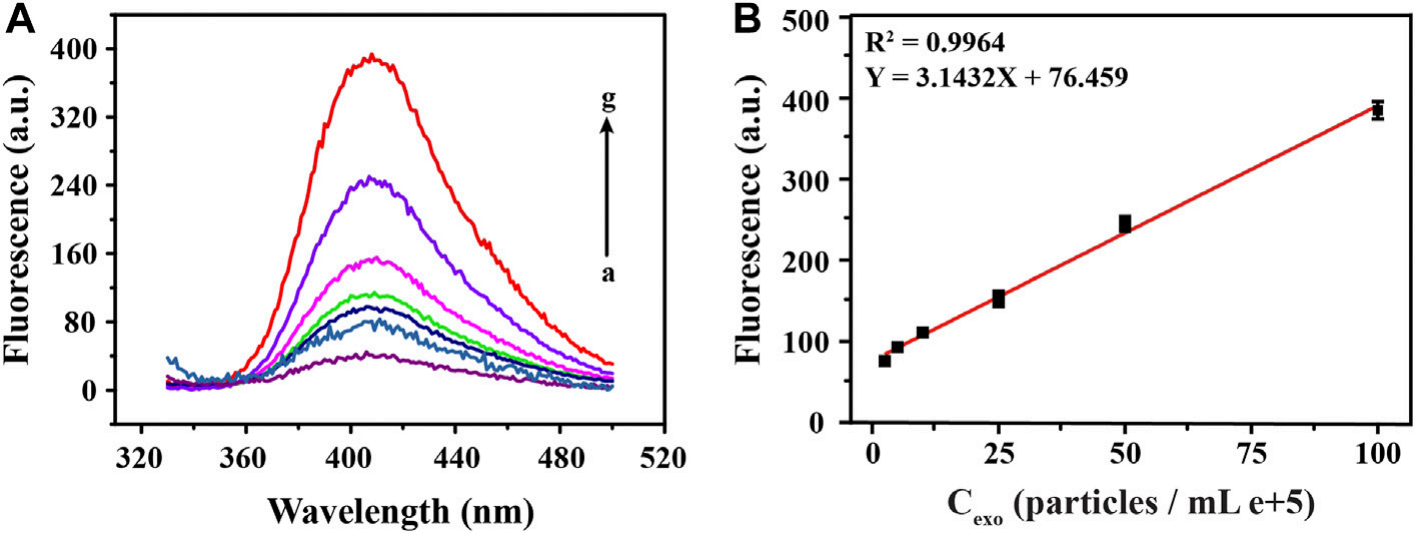
Sensitivity analysis of fluorescence detection of exosomes at different concentrations. The corresponding fluorescence emission spectra **(A)** and linear fittings **(B)** obtained by detecting a series of different concentrations of exosomes (0, 2.5, 5, 10, 25, 50, 100 particles/mL e+5, from a to g).

To explore the analytical performance of this method, we carried out linear research subsequently. As shown in Figure 4A, after plenty of different concentrations of exosomes were detected, a set of fluorescence signals was also obtained. Through analysis, we learned that, the intensity of the fluorescence signal was positively correlated with concentration of target exosomes. Moreover, Figure 4B was a linear fitting for the fluorescence signal obtained in Figure 4A and the corresponding exosomal concentrations, which shows that there was a good linear correlation between them. We obtained the regression equation between the exosome concentration (X) and corresponding fluorescence signal (Y) by linear fitting as Y = 3.1432 X + 76.459, with a correlation coefficient (*R* ^2^) of 0.9964. Then, according to the 3σ rule, which was defined as the mean value plus 3 times standard deviation of the blank (*n* = 3), the limit of detection (LOD) for exosomes was calculated to be 0.54 × 10^5^ particles/ml, comparable with other analytical methods (Karczmarczyk et al., 2016). Such a satisfactory detection performance was mainly attributed to the highly specific binding of the aptamer to the exosomal membrane protein and excellent catalytic performance of G4-hemin, which implies the rationality of the design for this strategy.

### Specificity Analysis

**FIGURE 5:**
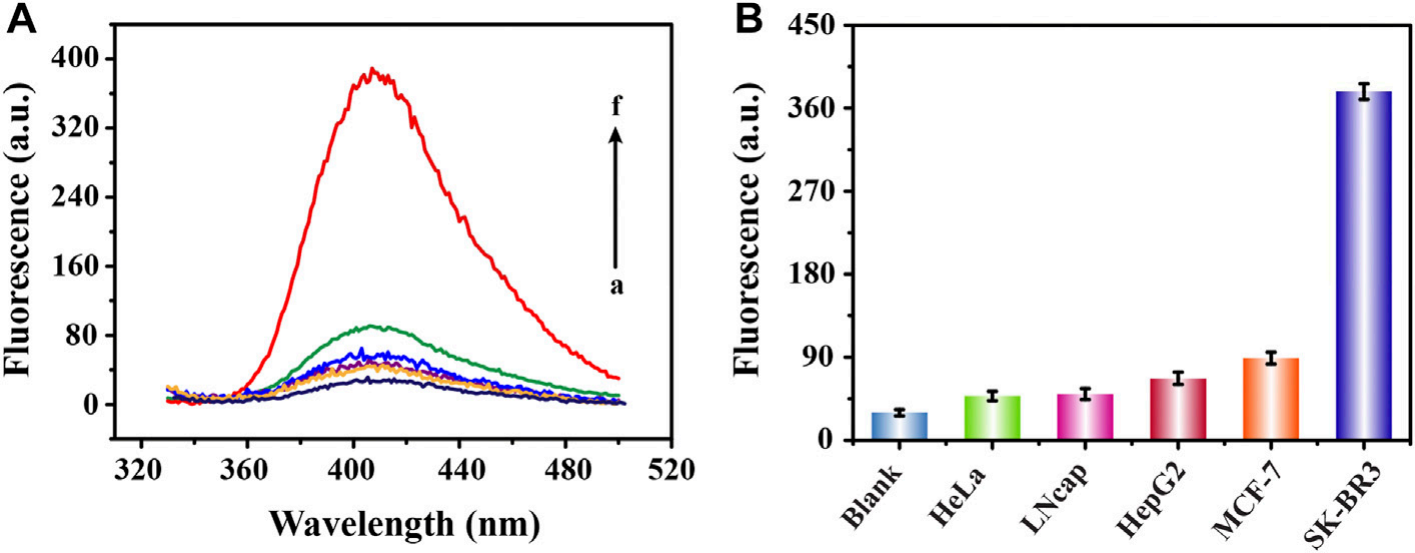
Specific analysis of fluorescence detection of exosomes derived from different cells. Fluorescence emission spectra **(A)** and corresponding signal response diagrams **(B)** obtained from the detection of five cell-derived exosomes (100 **×** 10^5^ particles**/**ml) (from b to f are HeLa, LNcap, HepG2, MCF-7 and SK-BR3 cell lines; a represents blank).

In order to prove the high specificity of this biosensing strategy in the detection of SK-BR3 derived exosomes, we selected five different cancer cell-derived exosomes for specificity analysis. We learned from Figure 5 that, compared with high fluorescence signal produced by SK-BR3 derived exosomes, the low signals of three lower-relevant cancer cell-derived exosomes (cervical cancer cells: HeLa, prostate cancer cells: LNcap and liver cancer cells: HepG2) were almost as negligible as the blank (Dahl et al., 2011; Liu et al., 2011; Feng et al., 2012). As another breast cancer cell of which expression of HER2 on the cell membrane is far less than that of SK-BR3, the signal obtained by MCF-7 cell-derived exosomes was slightly increased but was also far lower than that of SK-BR3 (Baumann et al., 2016). We also learned that, the affinity of aptamers was closely related to the expression of HER2 protein on exosome membrane. Such a high specific binding mode became a solid foundation for the successful release and assembly of G4-hemin as well as the subsequent display of catalytic performance.

### Detection of Exosomes in Clinical Samples

**FIGURE 6:**
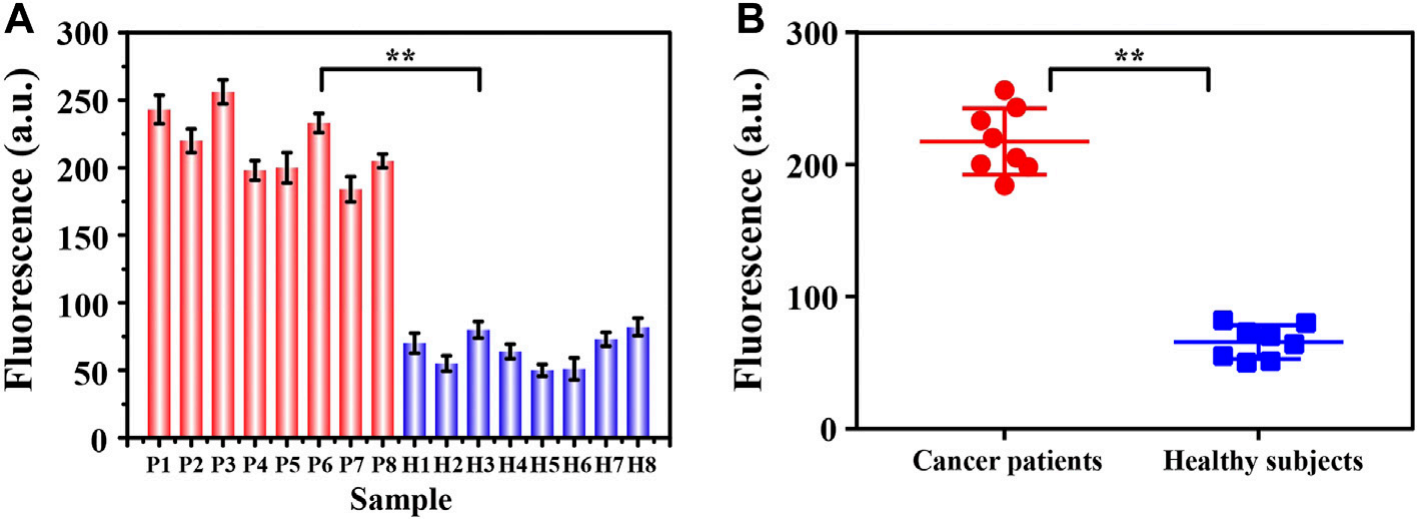
The signal intensity **(A)** and corresponding scatter plots **(B)** of exosomes from two groups of clinical samples (breast cancer patients: P1∼P8; healthy controls H1∼H8) were analyzed by this fluorescence sensing method. By Student’s unpaired *t*-test, the significance was determined. **p < 0.05.

Realizing the detection of trace targets in complex clinical samples is always the gold standard in judging whether a new method has the potential for clinical application. Inspired by this, we collected clinical serum samples for further analysis. As shown in Figure 6, we divided the collected samples into two groups, breast cancer patient group and healthy control group, each with eight samples. Apparently, we learned from the experimental results that the signals produced by the two groups of samples showed significant differences. The signals from the patient samples were all above 150 absorbance unit while the signals from the control group were all less than 100 absorbance unit, with relatively concentrated distribution. The statistically significant fluorescent signal difference between the two groups was learned easily from the scatter plot analysis in Figure 6B (*p* < 0.05), indicating the possibility for us to conduct preliminary screening of cancer patients through the intensity of the fluorescence signals. Aside from the simple and sensitive detection of exosomes, this method also has many notable advantages, including washing-free, time-saving, cost-effective and high repeatability. Furthermore, the detection of exosomes was completed with simple streamlined loading steps, which was easy to be transformed into clinical automated detection.

## Conclusion

Liquid biopsy is developing rapidly in tumor early diagnosis and therapeutic monitoring. While most research currently focuses on circulating tumor cells and circulating tumor DNA, exosomes and other extracellular vesicles are also emerging as a potentially broader and complementary platform for research. Exosomes contain a variety of substances derived from tumor cells, which has aroused great interest in the study of exosomes as tumor biomarkers. In order to solve the current limitations of breast cancer diagnosis, we proposed a washing-free, simple, and highly operable fluorescent biosensor based on an ingeniously designed bicyclic capture probe for detection of breast cancer cell-derived exosomes. The high efficiency and sensitivity of this detection method is attributed to the high affinity of aptamers and the excellent catalytic performance of G4-hemin. More importantly, the application of this fluorescent sensing strategy for detection of clinical samples was successful. And the preliminary testing results of cancer patients and non-cancer patients were quite different, which shows a good degree of discrimination. Based on the above, it is possible for us to conduct preliminary screening of breast cancer patients through the differentiated detection results. It is worth mentioning that by replacing aptamers, the proposed method is suitable for detecting more different types of cell-derived exosomes and their subpopulations, which has a certain application value. Moreover, due to the advantages of washing-free, easy-to-operate and high reproducibility, our novel detection method has the potential to be designed as a portable instrument for point of care test in the near future to simplify the clinical diagnosis process.

However, how to efficiently design and optimize the washing-free probe to achieve multiple detection of the target is the direction of our further research, which was also the limitation of this detection strategy. Hopefully, we will solve this problem in subsequent experiments and continuously improve the performance of the detection method.

## Data Availability

The original contributions presented in the study are included in the article/Supplementary Material, further inquiries can be directed to the corresponding authors.
