# Supplementary material for: A Washing-Free and Easy-to-Operate Fluorescent Biosensor for Highly Efficient Detection of Breast Cancer-Derived Exosomes
Source: Front Bioeng Biotechnol. 2022 Jun 28;10:945858. doi: 10.3389/fbioe.2022.945858 (PMC9273779; doi:10.3389/fbioe.2022.945858)
Supplement: Supplementary file 1 [file DataSheet1.docx]

**Supporting Information**

**A washing-free and easy-to-operate fluorescent biosensor for highly efficient detection of breast cancer-derived exosomes**

Wenqin Chen ^a, 1^, Yan Zhang ^a, 1^, Kaili Di ^a^, Chang Liu ^a^, Yanyan Xia ^a^, Shijia Ding ^b,^ ***, Han Shen ^a,^ **, Zhiyang Li ^a,^ *

^a^ *Department of Clinical Laboratory, Nanjing Drum Tower Hospital, The Affiliated Hospital of Nanjing University Medical School, Nanjing 210008, China*

*^b^ Key Laboratory of Clinical Laboratory Diagnostics (Ministry of Education), College of Laboratory Medicine, Chongqing Medical University, Chongqing 400016, China*

* Corresponding author.

** Corresponding author.

*** Corresponding author.

E-mail addresses: [dingshijia@163.com](mailto:dingshijia@163.com) (S. Ding); [shenhan10366@sina.com](mailto:shenhan10366@sina.com) (H. Shen); lizhiyangcn@qq.com (Z. Li).

^1^ Those authors contributed equally to this work.

**Table S1.** Sequences of oligonucleotides employed in this work.

| Oligonucleotides | Sequences (5’-3’) |
| --- | --- |
| Capture Probe | GTACTCGGGTGGGTGGGTGGGTCCACGCAGGGCCGTCGAACACGAGCATGGTGCGTGGACCTAGGATGACCTGAGTACTGTCC |

The blue part represents the G4 sequence, the yellow part represents the aptamer sequence, and the middle is the spacer sequence.

**Table S2.** Comparison of biosensing strategies for detecting exosomes.

| **Detection platform** | **Amplification strategy** | **Linear range (particles/mL)** | **LOD (particles/mL)** | **Ref.** |
| --- | --- | --- | --- | --- |
| Colorimetry | Enzyme-induced etching of gold nanobipyramid@MnO_2_ nanosheet nanostructures | 8.5 × 10^5^- 8.5 × 10^7^ | 1.35 × 10^5^ | [1] |
| Paper-based ITP technology | Isotachophoresis (ITP) technology | 1.2 × 10^6^- 2.0 × 10^6^ | 1.2 × 10^6^ | [2] |
| Fluorescence | Aptamer | 1.0 × 10^8^- 1.0 × 10^12^ | 1.0 × 10^8^ | [3] |
| Electrochemical | DNA nanotetrahedron coupled with enzymatic signal amplification | 2.16 × 10^4^- 7.5 × 10^7^ | 1.66 × 10^4^ | [4] |
| Fluorescence | Graphene oxide-DNA aptamer interactions | 3.0 × 10^7^ - 6.0 × 10^8^ | 2.1 × 10^7^ | [5] |
| Colorimetric | Aptasensor | 8.3 × 10^5^- 5.3 × 10^7^ | 3.94 × 10^5^ | [6] |
| Fluorescence | G4-hemin and aptamer | 2.5 × 10^5^ - 1.0 × 10^7^ | 0.54 × 10^5^ | This work |

**References**

1. Huang, R., He, L., Li, S., Liu, H., Jin, L., Chen, Z., et al. (2020). A simple fluorescence aptasensor for gastric cancer exosome detection based on branched rolling circle amplification. Nanoscale. 12, 2445-2451. doi: 10.1039/c9nr08747h.
2. Guo, S., Xu, J., Estell, A. P., Ivory, C. F., Du, D., Lin, Y., et al. (2020). Paper-based ITP technology: An application to specific cancer-derived exosome detection and analysis. Biosens. Bioelectron. 164, 112292. doi: 10.1016/j.bios.2020.112292.
3. Yu, X., He, L., Pentok, M., Yang, H., Yang, Y., Li, Z., et al. (2019). An aptamer-based new method for competitive fluorescence detection of exosomes. Nanoscale 11, 15589-15595. doi: 10.1039/c9nr04050a.
4. Li, Q., Wang, Y., Ling, L., Qiao, L., Chen, H., Ding, C., et al. (2021). Rapid and specific detection nanoplatform of serum exosomes for prostate cancer diagnosis. Microchim. Acta. 188, 283. doi: 10.1007/s00604-021-04934-7.
5. Wang, H., Chen, H., Huang, Z., Li, T., Deng, A., Kong, J. (2018). DNase I enzyme-aided fluorescence signal amplification based on graphene oxide-DNA aptamer interactions for colorectal cancer exosome detection. Talanta 184, 219-226. doi: 10.1016/j.talanta.2018.02.083.
6. Zhou, Y., Xu, H., Wang, H., Ye, B.C. (2019). Detection of breast cancer-derived exosomes using the horseradish peroxidase-mimicking DNAzyme as an aptasensor. Analyst. 14, 5107-5114. doi: 10.1039/c9an01653h.
